# Next generation sequencing of the clonal *IGH* rearrangement detects ongoing mutations and interfollicular trafficking in *in situ* follicular neoplasia

**DOI:** 10.1371/journal.pone.0178503

**Published:** 2017-06-22

**Authors:** Perikles Kosmidis, Irina Bonzheim, Claudia Dufke, Sema Colak, Thomas Hentrich, Christopher Schroeder, Peter Bauer, Patrick Adam, Falko Fend

**Affiliations:** 1 Institute of Pathology and Neuropathology, Eberhard Karls University of Tübingen and Comprehensive Cancer Center, University Hospital Tübingen, Tübingen, Germany; 2 Institute of Medical Genetics and Applied Genomics, University Hospital Tübingen, Tübingen, Germany; Institut Cochin, FRANCE

## Abstract

Follicular lymphoma (FL) is characterized genetically by a significant intraclonal diversity of rearranged immunoglobulin heavy chain (*IGH*) genes and a substantial cell migration activity (follicular trafficking). Recently, *in situ* follicular neoplasia (ISFN), characterized by accumulations of immunohistochemically strongly BCL2-positive, t(14;18)+ clonal B cells confined to germinal centers in reactive lymph nodes, has been identified as a precursor lesion of FL with low risk of progression to manifest FL. The extent of ongoing somatic hypermutation of rearranged IGH genes and interfollicular trafficking in ISFN is not known. In this study we performed an in depth analysis of clonal evolution and cell migration patterns in a case of pure ISFN involving multiple lymph nodes. Using laser microdissection and next generation sequencing (NGS) we documented significant intraclonal diversity of the rearranged IGH gene and extensive interfollicular migration between germinal centers of the same lymph node as well as between different lymph nodes. Furthermore, we identified N-glycosylation motifs characteristic for FL in the CDR3 region.

## Introduction

Follicular lymphoma (FL) is genetically characterized by the recurrent chromosomal translocation t(14;18)(q32;q21), present in the majority of cases [1]. The germinal center (GC) B-cell of origin of FL is subjected to ongoing somatic hypermutation (SHM) which results in an intraclonal sequence heterogeneity of neoplastic clones [2]. FL develops in the lymph node and infiltrates the bone marrow at an early time point of disease. Further migration of neoplastic cells between lymph nodes and bone marrow arises in both directions [3]. Due to the continuous exposure to the GC microenvironment and constitutive induced cytidine deaminase (AID) activity, a proportion of FL show increasing intraclonal diversity of rearranged immunoglobulin heavy chain (*IGH)* genes over time as evidence of SHM, whereas a second group of cases lacks intraclonal diversity compatible with clonal outgrowth [4]. Using *IGH* subclones as markers, cellular trafficking and dissemination based on phylogenetic relationships can be documented in FL. Substantial cell migration activity (follicular trafficking) has been described as a further hallmark of this lymphoma entity [3, 5–7].

*In situ* follicular neoplasia (ISFN) according to the recent update of the WHO classification, previously designated follicular lymphoma *in situ* (FLIS), is defined as a population of t(14;18)+, strongly BCL2 expressing clonal B cells strictly confined to germinal center structures of reactive lymph nodes [1]. ISFN was first described as *in situ* localization of follicular lymphoma in 2002 and can be identified as accumulations of strongly BCL2-protein and CD10 expressing B-cells with low proliferation rate in germinal centers of morphologically reactive lymph nodes, without alteration of architecture or extrafollicular spread neoplastic cells. These cells are clonal by immunoglobulin gene rearrangement analysis and carry the t(14;18)(q32;q21) translocation, the hallmark of manifest FL. Although ISFN may involve several lymph nodes as in the present case, it needs to be discerned from FL with partial involvement of lymph nodes, which shows at least focal disruption of architecture and extrafollicular spread of neoplastic cells and has a much higher frequency of progression than ISFN [8]. Similarly, ISFN in association with manifest FL does not simply represent initial involvement of germinal centers by the fully developed malignant clone. In summary, ISFN is thought to represent a putative precursor lesion for FL with low propensity for development of clinically manifest FL [9, 10]. We have recently corroborated this assumption, demonstrating through genetic analysis that (1) ISFN and matched manifest FL from the same patients were always clonally related and (2) that ISFN cells show significantly less secondary numerical aberrations as compared to manifest FL [10]. Although systematic studies are lacking, it is likely that only a minor part of the ISFN lesions progress to a manifest FL [11, 12]. In a recent study we detected prototypic ISFN lesions with a prevalence of 2.3% in reactive lymphoid tissues of individuals without a history or simultaneous presence of a manifest FL [13]. In part of these patients, ISFN lesions involved multiple adjacent germinal centers of the same node or even several lymph nodes without progressing to manifest FL [13]. This is in line with other studies demonstrating that for the majority of ISFN cases no evidence of FL was found at diagnosis or during follow-up [11, 14]. ISFN may represent an intermediate stage between manifest FL and rare t(14;18) positive B-cells which can be identified in the peripheral blood of approximately 50% of healthy adult individuals [15–17]. The prevalence of these cells increases with age, and individuals carrying a higher load of circulating t(14;18)+ have a 23-fold increased risk for the development of manifest FL [18, 19].

Although the role of ISFN as precursor lesion of FL with low incidence of transformation to manifest FL seems well established, the presence of intraclonal diversity and follicular trafficking, hallmarks of manifest FL, have not been investigated so far in ISFN lesions.

The goal of our study was therefore to use a next generation sequencing (NGS) approach to analyse the extent of SHM and migration patterns in a case of ISFN manifested in multiple germinal centers of several lymph nodes in an individual without history or presence of a manifest FL.

## Material and methods

### Case description

In a recent screening study we have identified cases of pure ISFN involving multiple lymph nodes (LN) without manifest FL by immunohistochemical staining reactive lymph nodes from unselected consecutive surgical specimens of patients without history of malignant lymphoma [13]. The case used for this study was one of the three identified ISFN cases in the previous study and derived from a 69-year-old man, who had undergone lobectomy for non-small cell lung cancer.

### Laser microdissection

For laser microdissection, 8 μm thick serial sections from each selected lymph node were cut. The first and every sixth slide were immunostained for the BCL2 protein to localize the ISFN lesions. The sections in between were H&E stained and not coverslipped for microdissection. Microdissection was performed using a Zeiss Axiovert 200 microscope and the P.A.L.M. system (Palm@Robo software V2.2; Zeiss Oberkochen, Germany). Only follicles with complete involvement by strongly BCL2 expressing B-cells were dissected and the laser beam was directed along the inner border of the germinal center, thus precluding contamination by reactive cells as much as possible.

### Polymerase chain reaction (PCR) of *IGH* rearrangements

DNA was extracted using proteinase K digestion followed by standard phenol/chloroform purification procedures [20]. Whole tissue sections without microdissection and microdissected samples with sufficient available DNA were subjected to a comparative clonality analysis. PCR for *IGH* framework 2 (FR2) gene rearrangements was performed as previously described using 0,5 U Phusion Hot Start DNA Polymerase (Finnzymes, Woburn, MA, USA) to detect the clonal nature of the ISFN lesion [21]. Modified amplification conditions were carried out with an initial denaturation step of 98°C (30 seconds), 40 cycles (98°C 10 seconds, 60°C 30 seconds, 72°C 30 seconds) and a final step of 10 minutes. The JH primer was modified with D4 fluorescent dyes (Sigma-Aldrich, St. Louis, MO, USA). For Genescan analysis 0.5 μl of the PCR products were mixed with sample loading solution containing 0.24 μl DNA size standard 400 (Beckman Coulter, Brea, CA, USA). The products were separated by capillary electrophoresis on the GenomeLab GeXP Genetic Analysis System and analyzed by the GenomeLab GeXP software 10.2 (Beckman Coulter, Brea, CA, USA). Additionally, single reactions with the respective V primer were performed to identify the rearranged V gene family. To confirm the rearranged V gene family, unlabelled PCR product (using the FR1 primer [21]) of the respective clonal population was enriched by agarose gel excision according to the base pair length, sequenced as previously described [22] and analyzed using IMGT-/V-Quest [23].

PCR products for next generation sequencing (NGS) analysis from microdissected follicles were generated using FR2 VH3 primers according to the modified BIOMED-2 protocol described for the GeneScan analysis above [21]. ISFN manifestations were dissected from serial sections of single follicles of one lymph node showing the most prominent lesions (eight samples), from multiple germinal centers of a region from the same lymph node pooled together (four samples) as well as from different single follicles of four further lymph nodes (four samples), resulting in altogether 16 samples.

### Preparation of samples for next generation sequencing on the Illumina Genome Analyzer IIx

PCR products of the 16 samples mentioned above were purified using AMPureXP magnetic beads (Beckman Coulter). In order to introduce Illumina Sequencing Adaptors as well as individual barcodes for each sample, a two-step PCR protocol was used. In the first PCR reaction the specific PCR products were re-amplified using composed primer pairs. The forward primer encompassed at the 5’ end the Illumina TruSeq Multiplexing specific adaptor sequence (containing the binding sequence for TruSeq read 1 sequencing primer) and at the 3’ end the VH3 FR2 primer sequence. The reverse primer consisted at the 5’ end of the Illumina Tru Seq multiplexing reverse adaptor sequence (containing the sequence for TruSeq multiplexing index-read and read 2 primers) and at the 3’ end of the JH consensus primer sequence. These adaptor primers were attached following a 10 cycle protocol at an annealing temperature of 60°C using Fast Start High Fidelity PCR System (Roche). Resulting PCR-products of expected fragment length were purified with AMPureXP as described above. P5 and P7 Sequences as well as barcodes for each sample were attached in a second PCR step. Since the Illumina multiplexing system provided 12 different barcode sequences, 4 barcode sequences had to be assigned twice. The barcode addition step was carried out using Phusion High Fidelity HF PCR MasterMix (New England Biolabs). In order to purify PCR products and completely deplete the samples from primer dimers, a stringent protocol for AMPureXP was used, removing fragments shorter than 300bp. For this purpose the barcoded PCR-products were mixed with an equal volume of AMPureXP.

Quantification and quality control of the samples was monitored on a DNA 100 Chip (Agilent 2100 Bioanalyzer, Agilent). Concentrations of the samples varied from 2.7 nM to 23.4 nM. Since four barcodes had been assigned to two samples each, two pools of samples had to be prepared. Pool #1, containing barcoded samples 1–12 was equimolar pooled to a concentration of 4 nM. Pool #2, containing samples 13–16 which were altogether higher in concentration (with barcodes 2, 4, 6 and 12) was equimolar pooled to 10nM. Following the Illumina Sequencing protocol, pools were denatured with 2 N NaOH, diluted to 6 pM and loaded on a flowcell in two different lanes. Cluster generation on the cBot (Illumina) as well as the 2x112bp +7 PE Sequencing run on the Genome Analyzer II (GAII, Illumina) was carried out according to manufacturer`s instructions. Coverage (total reads) per sample was between 143.320 and 30.528.

### Sequencing data analysis

After de-multiplexing of all samples with CASAVA v1.8 (Illumina), in-house scripts were used for primer trimming and quality filtering (mandatory per base quality above 20 on a “phred-like” scale for each base). High quality reads were converted to fasta-format and unique reads with a frequency below 0.1% were considered as noise and omitted. In order to bridge the gap in the FR3 part of the sequences arising through the determined read length from forward and reverse, 44 “N” bases were introduced. These unique sequences for each sample were submitted to IMGT/V-QUEST, an integrated alignment tool for nucleotide sequences of immunoglobulins and T-cell receptors [24]. The output format included the annotation of the V-, J- and D-genes and alleles, functionality, percentage of V-region identity, D- region reading frame, the amino acid sequence of the junction and the junction frame. The annotated unique sequences were then discriminated according to their similarity with the monoclonal reference sequence of the patient. Sequences differing profoundly with respect to V-, J- and D-genes and alleles as well as the junction were omitted from further analysis.

To further illustrate the genomic landscape, the selected unique sequences were submitted to ClustalW2 (http://www.ebi.ac.uk/Tools/phylogeny/clustalw2_phylogeny/) for phylogenetic analysis.

### Validation analyses of clonal *IGH* rearrangements

To validate sequencing of the clonal ISFN cells, six independently microdissected samples were analyzed (Foll. 1–26, Foll. 27–29, Foll. 30, LN14, LN15 and LN19) using a refined PCR approach in the NGS workflow (S1 File). In addition to evaluate the occurrence of potential PCR- or sequencing introduced errors, the diffuse large B cell lymphoma cell line HBL1 was sequenced using NGS. HBL1 was provided by Alexander Weber (Interfaculty Institute for Cell Biology, University of Tübingen, Germany), cultured [25] and processed to a formalin-fixed paraffin embedded cell block [26] as recently described. DNA isolation and NGS was performed the same way as with the ISFN samples obtained for validation.

### Ethical approval

The study was approved by the Local Ethics Committee of the Eberhard-Karls-University, Tübingen (300/210B01). The need for written informed consent from the patient was waived by the local Ethics Committee since the material was obtained more than five years ago.

## Results

### Histological and immunohistochemical findings

All 27 lymph nodes removed for staging of non-small lung cancer were morphologically free of carcinoma infiltrates and showed a normal architecture with variable amounts of reactive germinal centers, without evidence for FL (12). Immunostaining for BCL2 protein performed on all the nodes showed extensive ISFN lesions with typical intense positive staining of germinal centers of normal size with intact mantle zones in 16 of the 27 excised staging lymph nodes, without evidence for progression to manifest FL in any of the nodes. These germinal centers additionally showed strong CD10 positivity and a low proliferation rate in MIB1 staining. Fluorescence in situ hybridization demonstrated the presence of a break in the BCL2 locus indicative of a BCL2 translocation. The extension of the ISFN lesions ranged from single, isolated to several adjacent germinal centers per lymph node. Five of the 16 lymph nodes with the highest burden of ISFN cells were used for laser microdissection.

### Samples obtained for NGS

**Fig 1:**
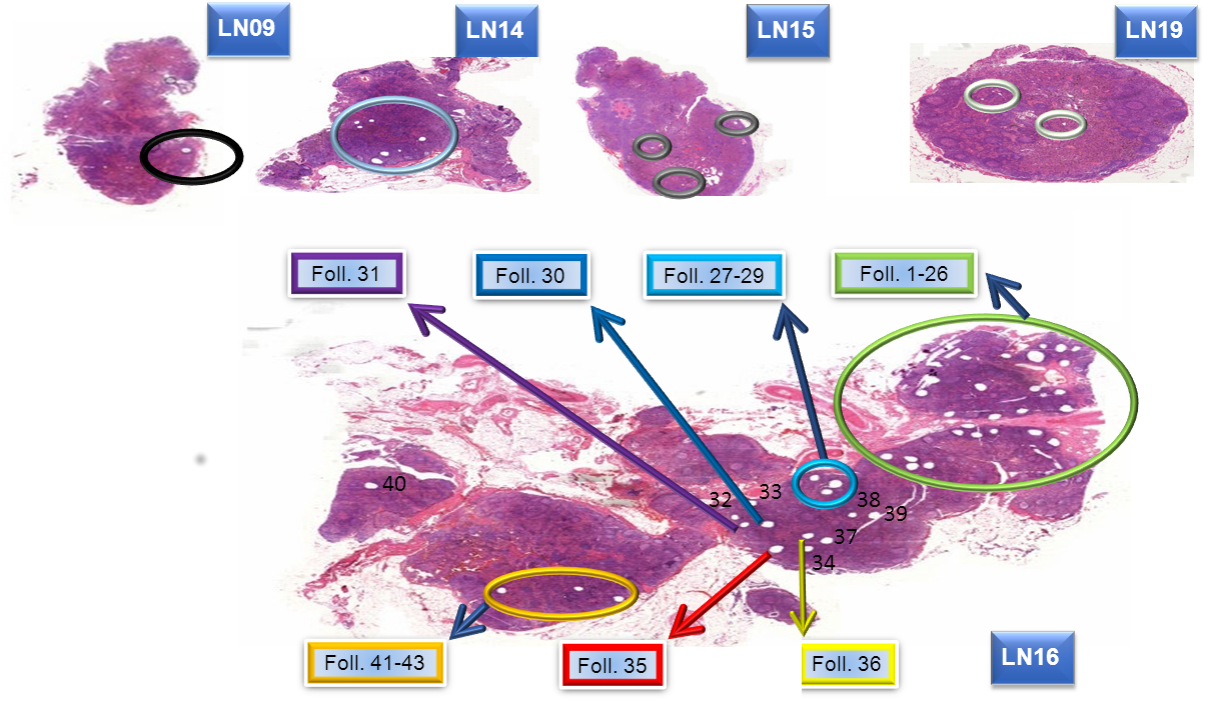
Overview of sample collection. H&E stained lymph nodes 9, 14, 15, 16 and 19 after microdissection with the highlighted areas from which the microdissections of ISFN-containing follicles were performed. The holes caused by microdissection are in part clearly visible.

In the first step, 16 DNA samples were obtained by microdissection for NGS analysis. Eight samples resulted from microdissection of single follicles (Foll. 30, Foll. 31, Foll. 35, Foll. 36; Foll. 37, Foll. 38, Foll. 39 and Foll. 40). Eight additional samples were gained either by pooling of several single microdissected follicles (Foll. 1–26, Foll. 27–29, Foll. 32–33 and Foll. 41–43) from lymph node 16, or all available ISFN-containing follicles of a single node were pooled in one sample (LN 9, 14, 15 and 19) (Fig 1).

Sequencing results of single follicles 30, 31, 35 and 36, pooled follicles 1–26, 27–29, and 41–43 (LN16), LN14, LN15 and LN19 were suitable for further analyses (see below), whereas single follicles 37, 38, 39, 40, pooled 32–33 and LN9 were excluded from further investigations as detailed below.

### Identification of the clonal *IGH* sequence

**Fig 2:**
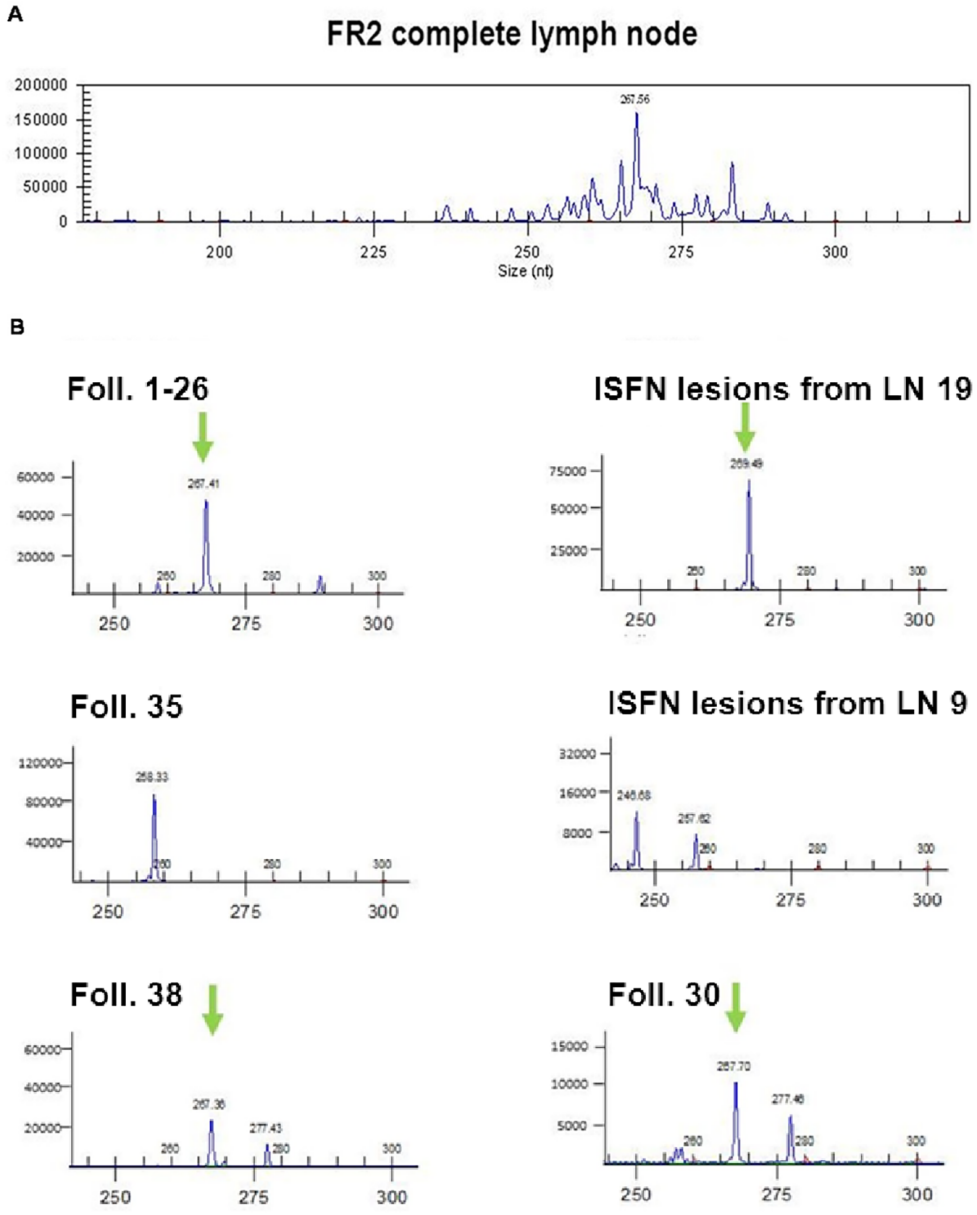
*IGH* clonality analysis of analysed follicles. A) Electropherograms of VDJ rearrangement clonality analysis using the framework 2 (FR2) primer set show a clonal product of 268 base pairs size. DNA from complete lymph node section. B) Electropherograms of six microdissected samples containing single or pooled ISFN lesions which showed products by fragment analysis based PCR amplification. Green arrows indicate the clonal 268 base pair products.

PCR based amplification of the *IGH* VDJ rearrangement using FR2 region primers and a JH primer from the whole tissue sections resulted in a reproducible peak of 268 bp with prominent polyclonal background in the GeneScan analysis (Fig 2A). The analysis of the microdissected tissue yielded a double peak of 265 and again 268 base pairs length. Sanger sequencing of the clonal product identified a productive V3-23 *IGH* gene rearrangement (D2-21/J4; S1 Fig). This sequence was subsequently identified in next generation sequencing (NGS) as read id 24. Eight of the ten additionally analyzed microdissected specimens with sufficient DNA for comparative clonality analysis showed a clonal product of 268 base pairs. In two of the evaluable specimens products of different length were detected, probably derived from contaminating B-cells (Fig 2B).

### NGS of the clonal *IGH* rearrangement

**Fig 3:**
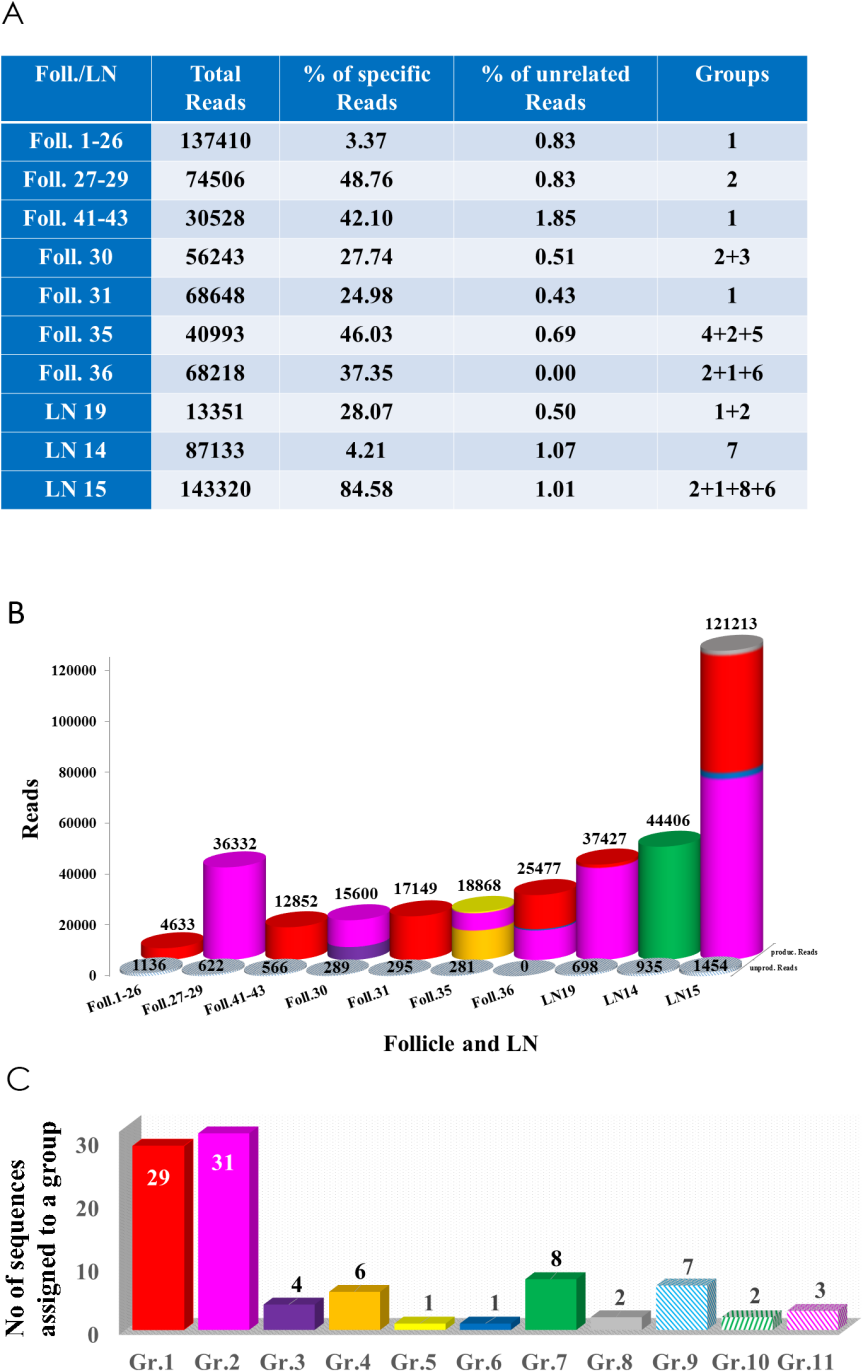
Identification of ISFN-associated reads and read groups. A) Table of the samples according to their total reads, percentage of specific and nonspecific reads and their groups. B) Numbers of specific reads which were identified in the different samples. Productive rearrangements are depicted as coloured bars indicating the assignment to the cluster groups; grey bars indicate unproductive rearrangements. In five samples no specific reads were identified. C) All specific reads clustered into 11 groups of identical CDR3 amino acid sequence based on sequence similarities. Columns indicate the number of respective sequences which are assigned to a group.

Deep sequencing and filtering of low-quality sequences of the 16 samples yielded from 13351 to 143320 sequence reads/sample, respectively (Fig 3A). Unique sequences were filtered according to the similarity of their CDR3-region compared to the monoclonal reference sequence (S1 Fig) previously generated by Sanger Sequencing of pooled DNA from all available microdissected follicles and were designated as clone-specific reads, comprising 25.4% of all specific *IGH* FR2 reads. Altogether 97 unique clone-specific sequences with similar CDR3 regions (S2 Fig) were available for characterization of the ISFN lesions. These amino acid sequence sequences showed very similar sequence motifs in their CDR3 regions (S3 Fig). The remaining 74.6% of total read sequences resembled productive *IGH* rearrangements, but CDR3 nucleotide and amino acid sequences did not show any similarity from the reference sequence and thus were regarded as being derived from contaminating reactive B-cells. The latter group of sequences was omitted from further analysis. The ISFN-specific reads showed a significant rate of point mutations (up to 12.7%) with clustering in the CDR2 and CDR3 region (S2 Fig).

Five of 16 samples were considered not evaluable concerning the clonal *IGH* rearrangements, since no ISFN-specific reads were detectable (samples from the single follicles, 38, 39 and 40, from the pooled follicles 32–33 and from the adjacent lymph node 9). One follicle (follicle 37) yielded only 645 (0.4%) ISFN-related reads (data not shown) and was also excluded from further analysis. The remaining 10 specimens contained between 3.4% and 85.6% clonal reads of the ISFN sequence, consisting of productive and unproductive rearrangements (0–19.7% unproductive rearrangements respectively) (Fig 3A and 3B).

The yield of reads corresponding to the clonal ISFN population varied considerably and showed an inverse correlation to the amount of dissected and pooled tissue, e.g. the single microdissected follicle 35 contained 46.7% relevant reads, whereas the analysis of follicles 1–26 pooled into a single sample yielded only 4.2% relevant reads, indicating a higher percentage of contaminating B-cells as could be expected (Fig 3B).

### ISFN cells show ongoing somatic hypermutation and signs of antigen selection

**Fig 4:**
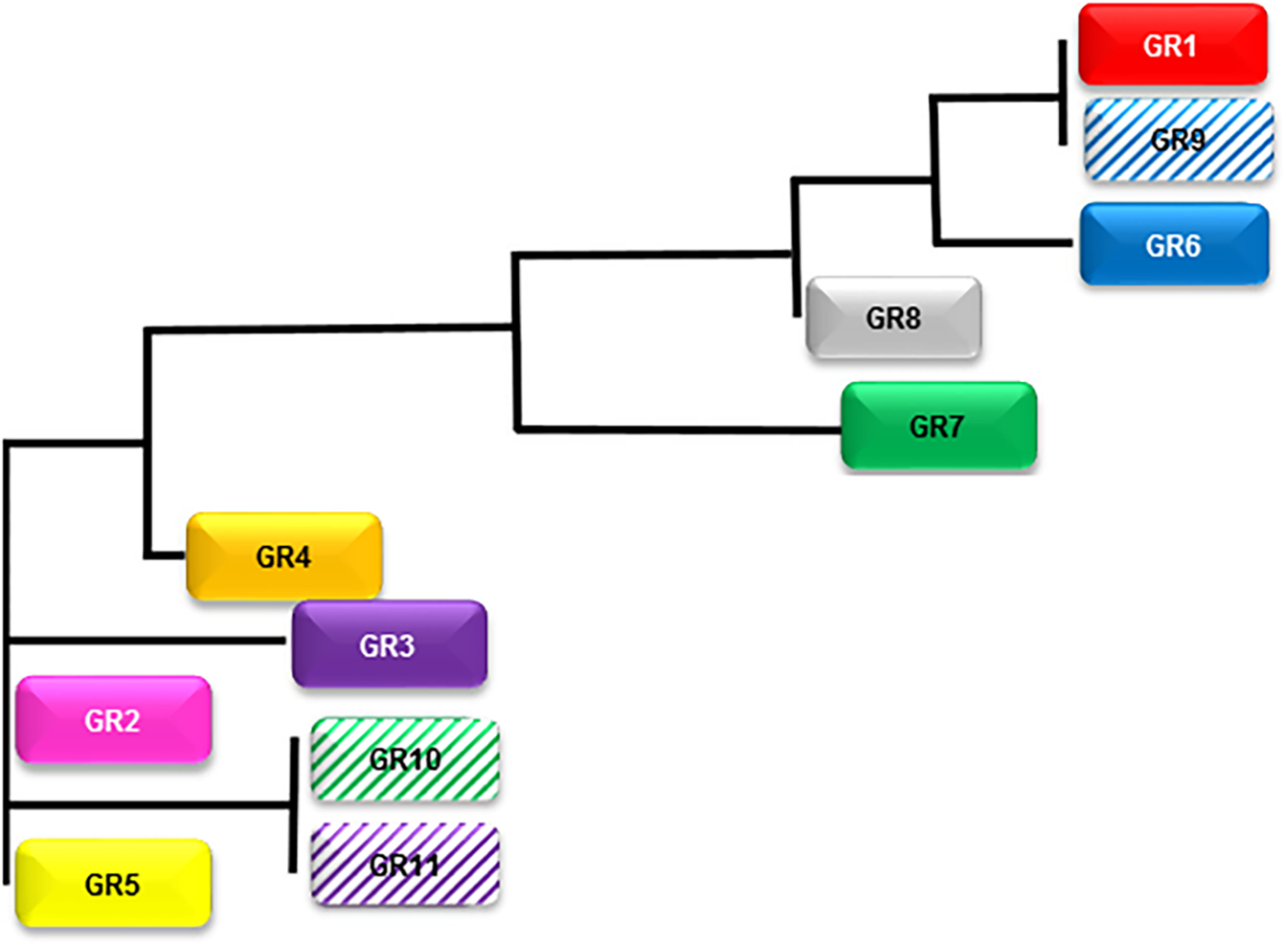
Phylogenetic tree of the eleven sequence groups. Spatial distance of the groups corresponds to the number of diverging base pairs. Dotted line indicates further distant relation to the three single sequences, which could not be assigned to the eleven groups.

The ISFN-specific sequences showed significant variations as evidence of ongoing somatic hypermutation. To follow interfollicular trafficking and dissemination of ISFN cells a phylogenetic tree was calculated based on the amino acid sequence similarities of the CDR3 region. The 95 relevant sequences could be assigned to eleven groups (G1-G11: 1–31 sequences per group) (Fig 3C), with three reads from follicle 37 not closely related to the eleven groups. The phylogenetic tree illustrates that the G3 group is genetically closest to the germ line sequence (alleles IGH V3-23, D2-21, J4) and therefore represents the origin of the genealogical tree of the tumor clone. The increasing number of additional mutations (V region identity/variation to germline sequence of the Sanger sequence: 87,5% (S1 Fig); spectrum of variations between Sanger sequence and ISFN-related reads 0,5–30% (1–55 mutations)) allowed the description of the genetic distance of the different groups of the phylogenetic tree and identified follicle 37 as being farthest from the ancestral clone (Fig 4, S3 and S4 Figs).

To exclude that the variations of the clonal ISFN sequence were due to PCR- or sequencing-introduced errors, validation was performed by sequencing independent microdissected samples. Of eight analyzed samples, in three an accumulation of clonal reads of G1 (24%, 94%) and G2 (14%) were detected (data not shown). The remaining samples only contained non-related sequences attributed to contaminating reactive B-cells. Additionally, NGS of DNA obtained from the formalin-fixed and paraffin-embedded HBL1 cell line resulted in 135928 reads which clustered in 9 read groups. 78% of reads showed an identical sequence, whereas the other 8 groups were separated only by single nucleotide substitutions (S5 Fig).

To see whether the ISFN cells showed signs of antigen selection, R/S mutation ratios were determined for the complete sequence between the CDR1 region and the JH segment obtained by Sanger sequencing derived from the whole section. The ratio of R/S mutations was 11 in the CDR regions, whereas in FR regions the ratio was 1.8 (data not shown). In addition, the eight NGS sequence groups showing productive rearrangements contained the previously described structural feature of FL cells, a sequence motif which acts as acceptor sites for N-addition of glycan chains (Asn-X-Ser/Thr) (S6 Fig) [27].

### ISFN cells show significant interfollicular migration

Topographic allocation of sequence cluster groups to the dissected follicle structures exhibited significant interfollicular trafficking. Representatives of the different sequence cluster groups were detected with varying frequency not only in adjacent follicular structures but also in different lymph nodes, with G2 followed by G1 detected with the highest frequency. Reads of both clusters were found in adjacent follicles as well as in different lymph nodes, whereas clusters G5, G6 and G8 were restricted to a sole localization.

## Discussion

As a germinal center neoplasm, FL is subjected to ongoing somatic hypermutation of the rearranged immunoglobulin genes, which can be used to delineate clonal evolution and migration patterns of neoplastic cells. In this study of a case of ISFN with multiple involved lymph nodes, we demonstrate that the hallmarks of FL, ongoing somatic hypermutation and interfollicular trafficking are readily detectable in this pre-clinical stage of a genetically less advanced FL precursor.

These data corroborate the hypothesis that ISFN is the first identifiable FL precursor lesion in tissue context and represents the putative link with clonal B-lymphocytes carrying the chromosomal translocation t(14;18)(q32;q21) in the peripheral blood of healthy adult individuals. Although these cells were originally believed to belong to the naïve B cell subset, it was demonstrated more recently, that in fact the t(14;18) translocation is carried by low-affinity memory B cells of GC origin, sharing genotypic and phenotypic features with FL and prone to constitute a premalignant FL niche [28, 29]. It was suggested that these t(14;18)-positive cells proliferate in response to antigens and that persistent antigenic stimulus is necessary for maintenance of these clones, requiring repeated cycles of GC re-entry. In addition to continuous BCR signalling, the acquisition of acceptor sites for N-addition of glycan chains (Asn-X-Ser/Thr) could result in alternative mechanisms of survival signalling through lectin-mediated interactions with the local environment or bacterial antigens [27, 30–32]. However, experimental evidence from tissue-based studies supporting this hypothesis have been largely lacking so far.

In our study, using a combination of conventional sequencing for identification of the dominant clonal *IGH* rearrangement, next generation sequencing to track somatic hypermutation, and microdissection to analyse topographically distinct regions we were able to document significant clonal evolution, as well as trafficking of ISFN cells between involved nodes. “Ongoing” mutations served to perform a phylogenetic analysis. The detected ISFN *IGVH* sequences clustered in eight genetic clusters with productive rearrangements and 3 clusters with unproductive rearrangements consisting each of 1–33 different sequences detected with different incidence rates in the elven samples analyzed. Since we worked with routinely formalin-fixed and paraffin-embedded archival tissues, demonstration that the observed variability of the clonal sequence truly represents clonal evolution rather than technical artifacts is of crucial importance. We therefore used two different strategies for validation of our results. Firstly, by performing NGS of the rearranged *IGH* gene of the DLBCL cell line HBL1 lacking ongoing mutations for comparison, we could demonstrate that even after paraffin embedding only very few single substitutions arise in a minority of reads which probably represent artifacts. As mainly G>A or C>T transitions were detected, it can be assumed that these artifacts are the result of formalin fixation which promotes deamination of cytosine residues [33]. Since the frequency of base changes observed in the ISFN was several-fold higher compared to the HBL1 cell line, we assume that at least the majority of them represent true clonal evolution. Furthermore, sequencing of independently microdissected involved follicles again identified the most prominent read groups G1 and G2, confirming the dissemination of the clonal ISFN cells, and validated the detection of ongoing mutations. As expected, unproductive clonally related reads were also identified in the different samples, albeit at lower numbers, possibly reflecting a disadvantage for the B-cell carrying this V_H_ sequence. By mapping the different sequences back to the microdissected areas, we were able to show a significant amount of migration between different follicles and lymph nodes.

These findings have several implications for our understanding of the precursor stages of FL. In line with a case report which demonstrated clonal relationship between circulating FL-like B-cells and ISFN in a single patient [34], our finding of clonal B-cells with an identical or closely related position on the genealogical tree in different lymph nodes support the notion that ISFN cells can migrate and thus alternate between tissue and peripheral blood. This indicates that ISFN is not a locally restricted clonal expansion, but rather a tissue stage of preneoplastic t(14;18)+ B-cells possibly induced to accumulate locally by an antigenic stimulus [12, 34]. On the other hand, the varying proportions of different sequence groups in the investigated areas, with some clones restricted to a single area may indicate that single colonized germinal centers in ISFN seeded by circulating FL-like B-cells may exhibit local clonal expansions not apparent in other areas, although our study is probably not extensive enough to reveal the full scope of clonal evolution and interfollicular and internodal trafficking. Nonetheless, the presence of significant follicular trafficking in ISFN is similar to manifest FL [5], whereas the lymphocyte migration rate between different germinal centers is low under physiological conditions in non-neoplastic lymph nodes [35].

Another interesting finding of our study is the fact that all eight cluster groups showing the same productive CDR3 amino acid sequences also contained the sequence motif N-X-S/T, which previously had been shown as a characteristic of FL, which can serve as glycosylation sites [27, 30–32, 36]. This finding is in line with a recent case study of ISFN and clonally related manifest FL, which identified N-glycosylation motifs in the rearranged sequence, indicating that already at this early stage of FL development, mechanisms to bypass the need of a high affinity BCR are already active and may secure the long-term survival of the clone [37]. This further supports the active role of the germinal center microenvironment in the preservation and perhaps expansion of the premalignant clone and could indicate that immune processes resulting in the formation of reactive germinal centers in regional nodes may provide a homing ground for circulating t(14;18)+ cells.

Although we were not able to sequence the whole rearranged *IGHV* sequence from the microdissected follicles for technical reasons, NGS analysis detected an accentuation of point mutations in the CDR2 and CDR3 regions with a lower mutation rate in the FR regions as an indication for antigen selection. The same was true when we analyzed the whole *IGVH* sequence obtained from Sanger sequencing of enriched, but not laser-microdissected ISFN. In addition, similar to previous investigations of manifest FL, the R/S ratio in the FR regions was below 2.845, which is the inherent mean value of the R/S ratio for human germline V_H_ genes [38]. This indicates that the structure of the BCR is preserved irrespective of the total number of somatic mutations and suggests that a functional BCR has been important for cell survival during the early stages of clonal evolution [2]. Another potential selection mechanism discussed by Zuckerman et al. is selection of mutated IGV sequences towards preservation of the structural integrity rather than specific antigen binding [36].

In summary, our study documents for the first time significant intraclonal diversity and extensive interfollicular and internodal migration of preneoplastic precursor cells of FL on the tissue level and indicates, that the mechanisms of clonal evolution of the rearranged V_H_ gene are already active in ISFN. Further studies are warranted to elucidate the mechanisms of progression of ISFN on the genetic level.

## Supporting information

S1 Fig Sanger sequencing of the clonal ISFN IGH product. DNA of microdissected follicles was amplified using V_H_3-FR1 and J_H_ consensus primers as previously described [21] and subjected to Sanger sequencing to confirm the clonal V3 rearrangement detected by GeneScan analysis. A productive *IGH* rearrangement V3-23/D2-21/J4 was identified using IMGT/V-QUEST [24].
(TIF)

S2 Fig Alignment of all ISFN-specific reads. First sequence is the Sanger sequence of pooled DNA from all available follicles, which was used to identify specific reads. Framed nucleotides indicate somatic hypermutations.
(TIF)

S3 Fig Phylogenetic tree of sequence groups based on amino acid sequence similarities of the CDR3 region. Phylogenetic tree of the eleven sequence groups showing the amino acid sequence of the CDR3 regions (calculated amino acids sequences are modified from IMGT/V-QUEST [24]). Colored labels were assigned to each group and groups of unproductive rearrangements labels are striped.
(TIF)

S4 Fig Phylogenetic tree of read sequences of the ISFN clone. Phylogenetic tree of the 97 specific read sequences. Colored labels were assigned to each group and groups of unproductive rearrangements are shaded. Double crosses indicate further distant relations.
(TIF)

S5 Fig Alignment of *IGH* rearrangement sequences of the cell line HBL1. NGS of the HBL1 cell line yielded 135928 reads which clustered in 9 read groups. A minority of sequences (22%) from eight groups showed single substitutions in comparison to the first group of identical sequences (78%).
(TIF)

S6 Fig Identification of glycosylation sites. Amino acid sequences of the CDR3 region of the eight cluster groups which were composed of productive rearrangements. The frame indicates the sequence motif which acts as acceptor site for N-addition of glycan chains (Asn-X-Ser/Thr).
(TIF)

S1 File Supporting methods. (DOCX)
