# Supplementary figures and images for: Next generation sequencing of the clonal IGH rearrangement detects ongoing mutations and interfollicular trafficking in in situ follicular neoplasia
Source: PLoS One. 2017 Jun 22;12(6):e0178503. doi: 10.1371/journal.pone.0178503 (PMC5480878; doi:10.1371/journal.pone.0178503)

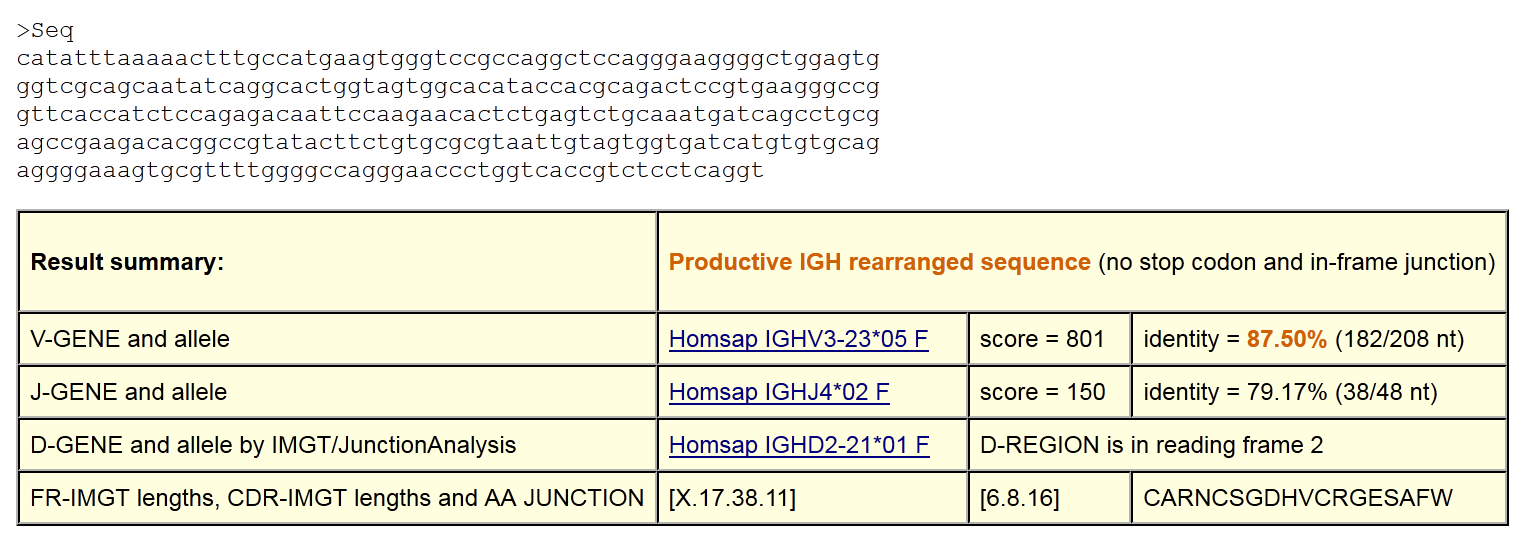

Supplement: S1 Fig — DNA of microdissected follicles was amplified using VH3-FR1 and JH consensus primers as previously described [21] and subjected to Sanger sequencing to confirm the clonal V3 rearrangement detected by GeneScan analysis. A productive IGH rearrangement V3-23/D2-21/J4 was identified using IMGT/V-QUEST [24]. (TIF) [file pone.0178503.s001.tif]

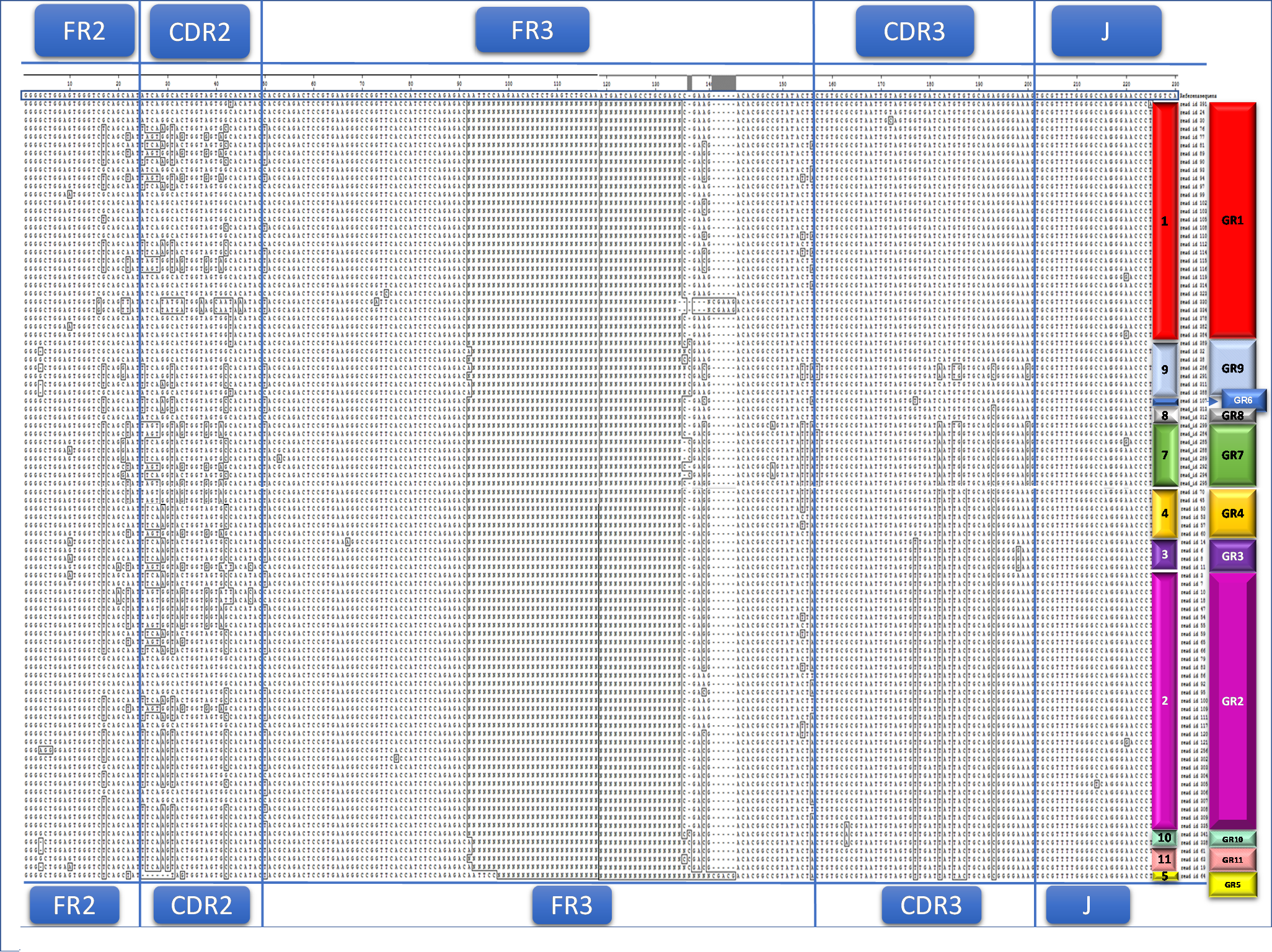

Supplement: S2 Fig — First sequence is the Sanger sequence of pooled DNA from all available follicles, which was used to identify specific reads. Framed nucleotides indicate somatic hypermutations. (TIF) [file pone.0178503.s002.tif]

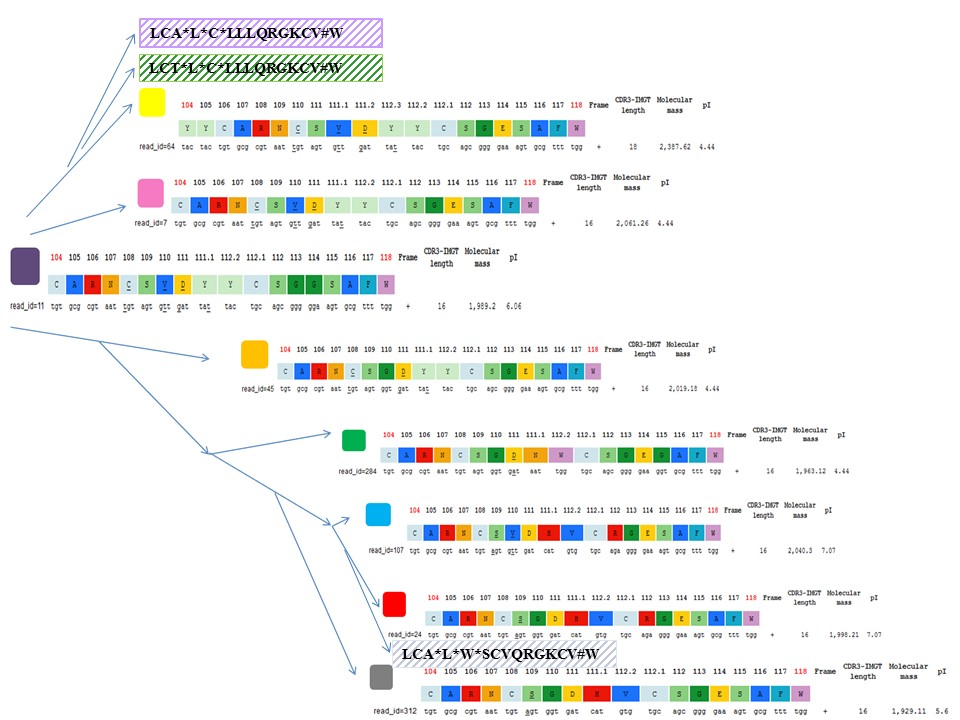

Supplement: S3 Fig — Phylogenetic tree of the eleven sequence groups showing the amino acid sequence of the CDR3 regions (calculated amino acids sequences are modified from IMGT/V-QUEST [24]). Colored labels were assigned to each group and groups of unproductive rearrangements labels are striped. (TIF) [file pone.0178503.s003.tif]

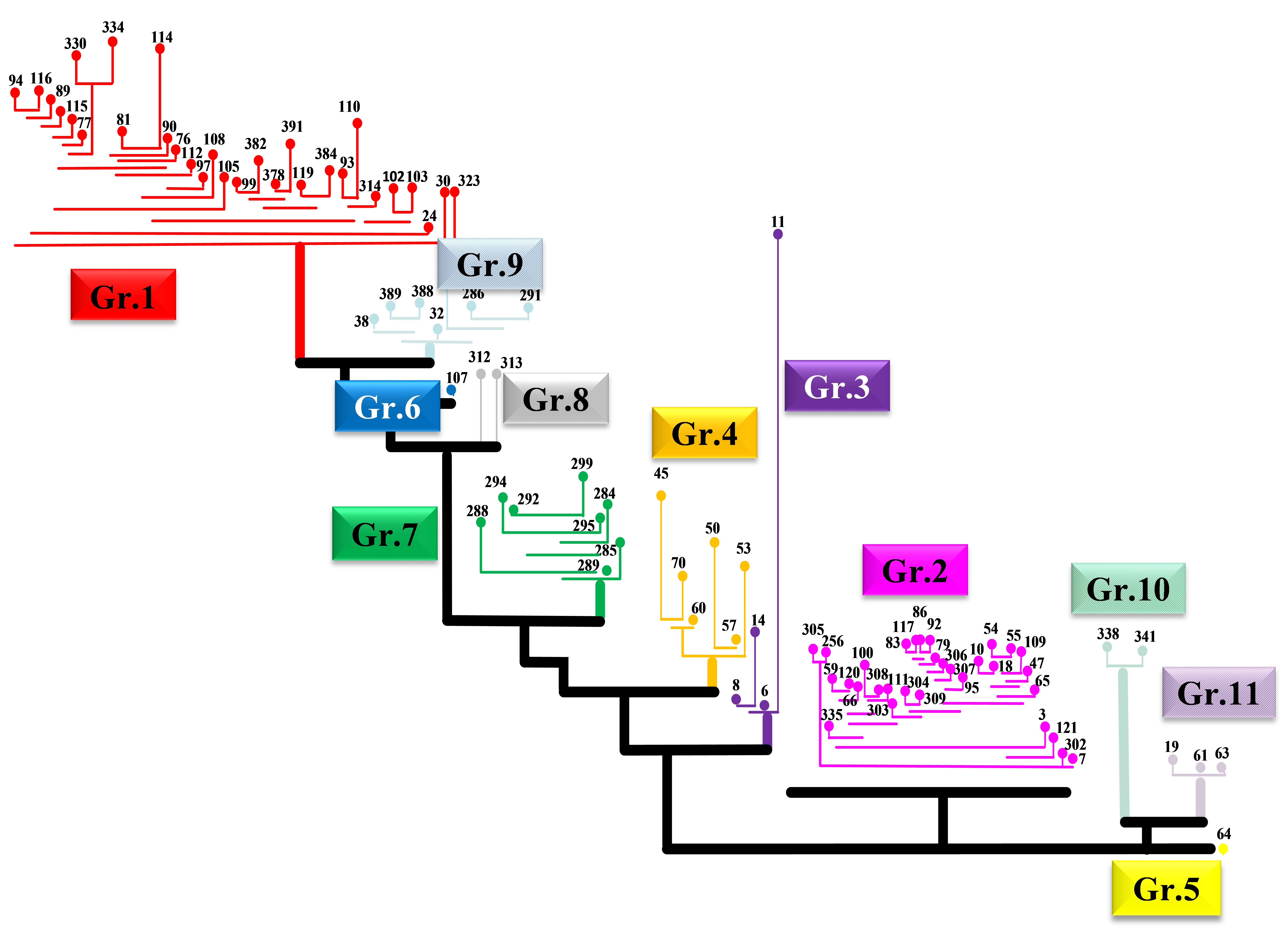

Supplement: S4 Fig — Phylogenetic tree of the 97 specific read sequences. Colored labels were assigned to each group and groups of unproductive rearrangements are shaded. Double crosses indicate further distant relations. (TIF) [file pone.0178503.s004.tif]

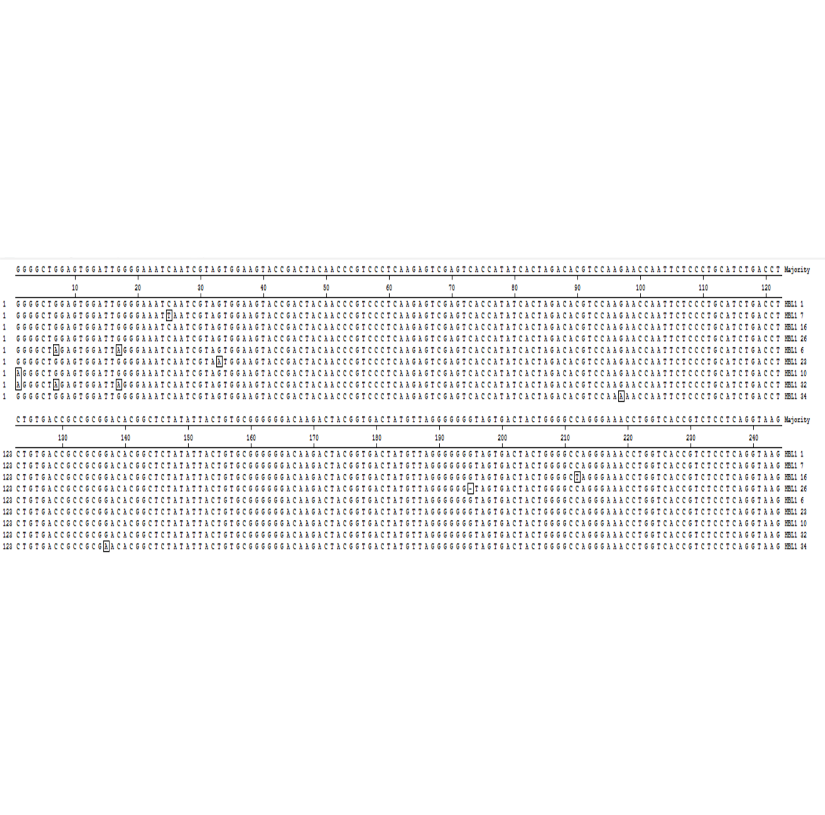

Supplement: S5 Fig — NGS of the HBL1 cell line yielded 135928 reads which clustered in 9 read groups. A minority of sequences (22%) from eight groups showed single substitutions in comparison to the first group of identical sequences (78%). (TIF) [file pone.0178503.s005.tif]

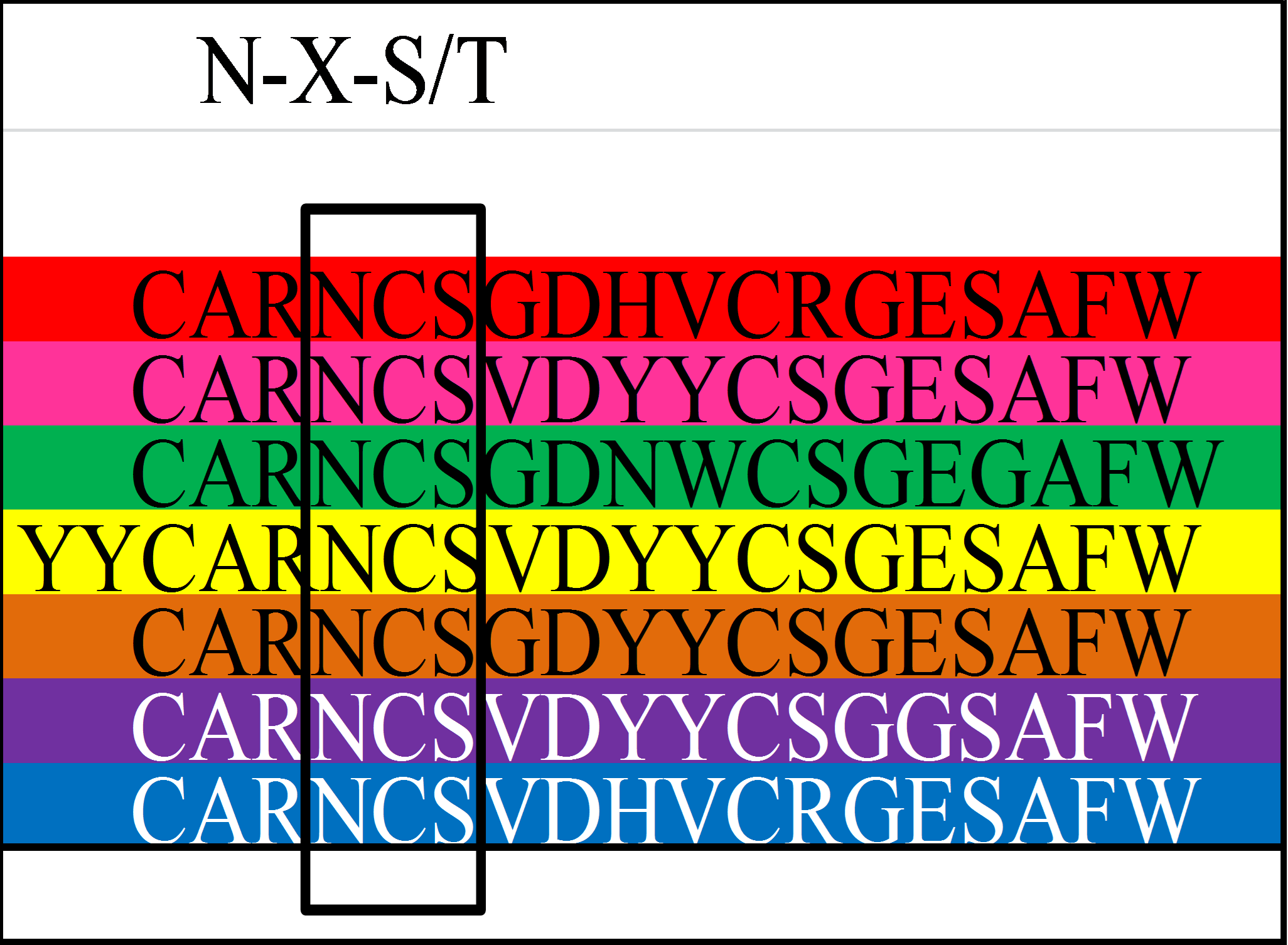

Supplement: S6 Fig — Amino acid sequences of the CDR3 region of the eight cluster groups which were composed of productive rearrangements. The frame indicates the sequence motif which acts as acceptor site for N-addition of glycan chains (Asn-X-Ser/Thr). (TIF) [file pone.0178503.s006.tif]
