# Supplementary material for: Next generation sequencing of the clonal IGH rearrangement detects ongoing mutations and interfollicular trafficking in in situ follicular neoplasia
Source: PLoS One. 2017 Jun 22;12(6):e0178503. doi: 10.1371/journal.pone.0178503 (PMC5480878; doi:10.1371/journal.pone.0178503)
Supplement: S1 File — (DOCX) [file pone.0178503.s007.docx]

**Supporting Information**

**S1 File: Supporting methods**

**Validation analyses of clonal *IGH* rearrangements**

PCR products of *IGH* rearrangements of eight independently microdissected samples (Foll. 1-26, Foll. 27-29, Foll. 30, Foll.32-33, Foll. 40, LN14, LN15 and LN19) were generated using Fluidigm-tailed primers (Fluidigm_F: 5’-ACACTGACGACATGGTTCTACA-3‘ and Fluidigm_R: 5‘-TACGGTAGCAGAGACTTGGTCT-3’) with the BIOMED-2 protocol [1]. PCR products were subsequently purified using Agencourt AMPure XP reagent (Beckman Coulter, Brea, CA, USA) according to the manufactureres manual. In a second PCR step NGS sequencing adapters and barcodes (Barcode Sequences for Access Array Barcode Library for Illumina 'Sequencers- 384, Fluidigm, South San Francisco, CA, USA) were added to the PCR products. PCR was performed using 1 µl of 1:10 diluted first PCR product in a final volume of 20 µl with 0.2 µM of each primer, 0,2 mM dNTPs, 1.8 mM MgCl2 and one Unit Fast Start High Fidelity Enzyme Blend (Roche, Rotkreuz, Schweiz). Cycling conditions entailed an initial denaturation at 95°C for 10 min followed by 10 cycles of denaturation (95°C for 15 sec), annealing (60°C for 30 sec) and elongation (72°C for 60 sec), with a ﬁnal elongation at 72°C for 3 min. PCR products were purified a second time using Agencourt AMPure XP reagent. Using different barcodes the samples were equimolarly pooled to 14 nM.

Sequencing was performed on a MiSeq (Illumina, San Diego, CA, USA) in paired-end mode according to the manufacturer’s protocol (MiSeq V2 chemistry 400 cycles). Sequencing data analysis was done as described in the methods part of the article.

**S1 Fig:**

**Sanger sequencing of the clonal ISFN IGH product.**

DNA of microdissected follicles was amplified using V_H_3-FR1 and J_H_ consensus primers as previously described [1] and subjected to Sanger sequencing to confirm the clonal V3 rearrangement detected by GeneScan analysis. A productive *IGH* rearrangement V3-23/D2-21/J4 was identified using IMGT/V-QUEST^®^ [2].

**S2 Fig:**

**Alignment of all ISFN-specific reads.**

First sequence is the Sanger sequence of pooled DNA from all available follicles, which was used to identify specific reads. Framing of nucleotides indicate somatic hypermutations.

**S3 Fig:**

**Phylogenetic tree of sequence groups based on amino acid sequence similarities of the CDR3 region.**

Phylogenetic tree of the eleven sequence groups showing the amino acid sequence of the CDR3 regions (calculated amino acids sequences are modified from IMGT/V-QUEST^®^ [2]). Colored labels were assigned to each group and groups of unproductive rearrangements labels are striped.

**S4 Fig:**

**Phylogenetic tree of read sequences of the ISFN clone.**

Phylogenetic tree of the 97 specific read sequences. Colored labels were assigned to each group and groups of unproductive rearrangements are shaded. Double crosses indicate further distant relations.

**S5 Fig:**

**Identification of glycosylation sites.**

Amino acid sequences of the CDR3 region of the eight cluster groups which were composed of productive rearrangements. Framing indicates the sequence motif which acts as acceptor site for N-addition of glycan chains (Asn-X-Ser/Thr).

1. van Dongen JJ, Langerak AW, Bruggemann M, Evans PA, Hummel M, Lavender FL, et al. Design and standardization of PCR primers and protocols for detection of clonal immunoglobulin and T-cell receptor gene recombinations in suspect lymphoproliferations: report of the BIOMED-2 Concerted Action BMH4-CT98-3936. Leukemia. 2003;17(12):2257-317. Epub 2003/12/13. doi: 10.1038/sj.leu.2403202

2403202 [pii]. PubMed PMID: 14671650.

2. Brochet X, Lefranc MP, Giudicelli V. IMGT/V-QUEST: the highly customized and integrated system for IG and TR standardized V-J and V-D-J sequence analysis. Nucleic Acids Res. 2008;36(Web Server issue):W503-8. Epub 2008/05/27. doi: gkn316 [pii]

10.1093/nar/gkn316. PubMed PMID: 18503082; PubMed Central PMCID: PMC2447746.
